# THE SOCIOCULTURAL MODEL OF CREATIVITY: HOW IT CAN EXPLAIN THE SIGNIFICANCE OF MULTIPLE FORMS OF LATE-LIFE CREATIVITY

**DOI:** 10.1093/geroni/igz038.1555

**Published:** 2019-11-08

**Authors:** Carolyn E Adams-Price

**Affiliations:** Department of Psychology Mississippi State University, Starkville, Mississippi, United States

## Abstract

Creativity in later life comes in many forms, ranging from everyday creativity to genius-level creativity, and including both newly learned creative activities and life-long creative hobbies. Previous psychosocial models of creativity have had limited utility in explaining the significance of late life creativity. Glaveanu’s sociocultural model has not been previously applied to older adults, but its inclusiveness makes it supremely useful for describing the range of creative activities and products in context. Creativity, according to Glaveanu, involves five interconnected components: actors, actions, artifacts, audiences, and affordances, which can be used to describe many different points along continuum of creative activities. The sociocultural model recognizes the value of different levels of creativity, including culturally-specific crafts, practice and learning new skills, the role of large and small audiences for creativity, and the connection of creativity and community. Glaveanu’s model will be discussed in the context of Erikson’s theory of late life development.

